# Supplementary material for: Efficient Neutrophil Activation Requires Two Simultaneous Activating Stimuli
Source: Int J Mol Sci. 2021 Sep 18;22(18):10106. doi: 10.3390/ijms221810106 (PMC8467451; doi:10.3390/ijms221810106)
Supplement: Supplementary file 1 [file ijms-22-10106-s001.zip › ijms-1387780-supplementary.pdf]

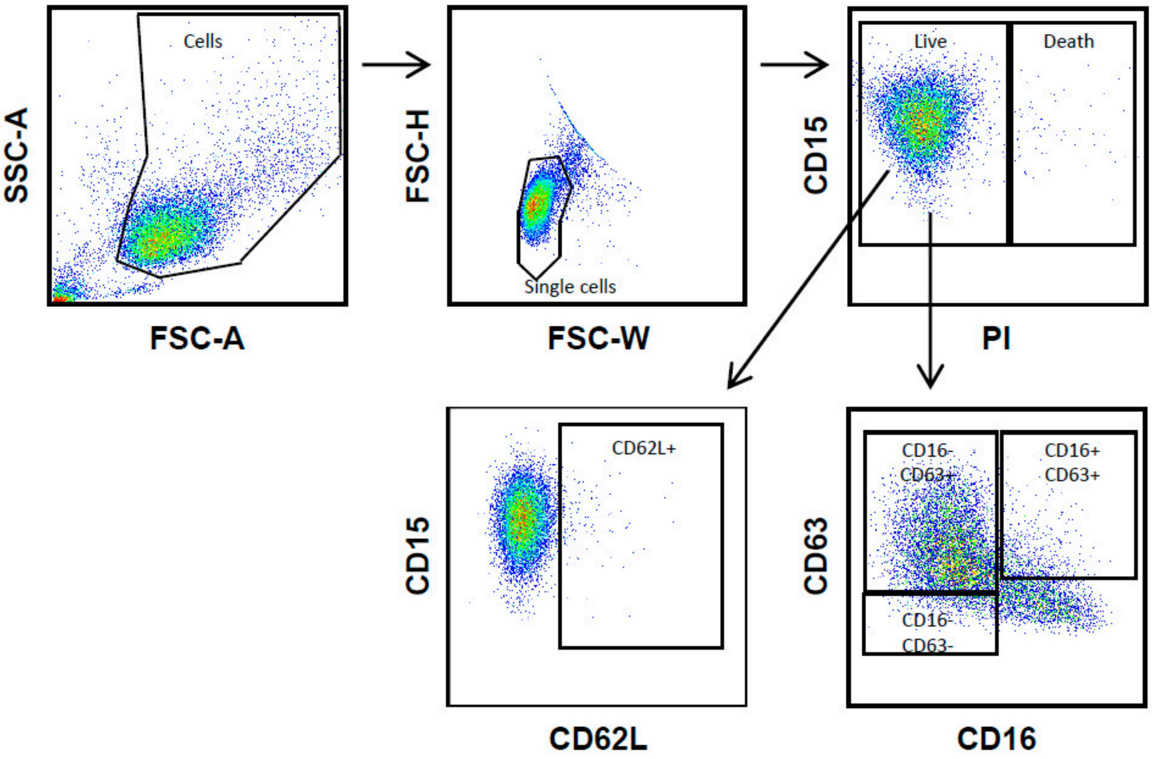

Figure S1. Flow cytometry gating strategy for neutrophil degranulation. Neutrophils were first gated on forward scatter(FSC-A) and side scatter (SSC-A) plot. Then single cells were gated and then live and death cells were gated. With the live cells gates were set for activation markers.

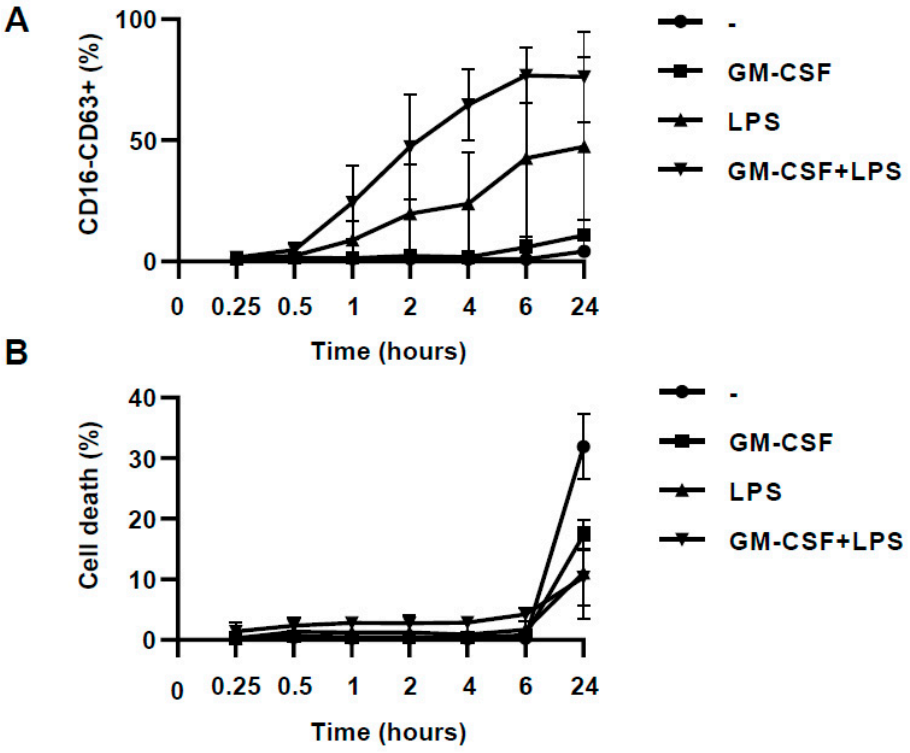

Figure S2. Neutrophils were cultured in the absence or presence of GM-CSF (50 U/mL), LPS (10 ng/mL), or their combination. After different culture durations flow cytometry was used to asses activation status. (**A**) Full degranulation over time, measured by percentages of CD16-CD63+ neutrophils. (**B**) Cell death over time (n = 3–5 per time point). Data are presented as mean ± SD.

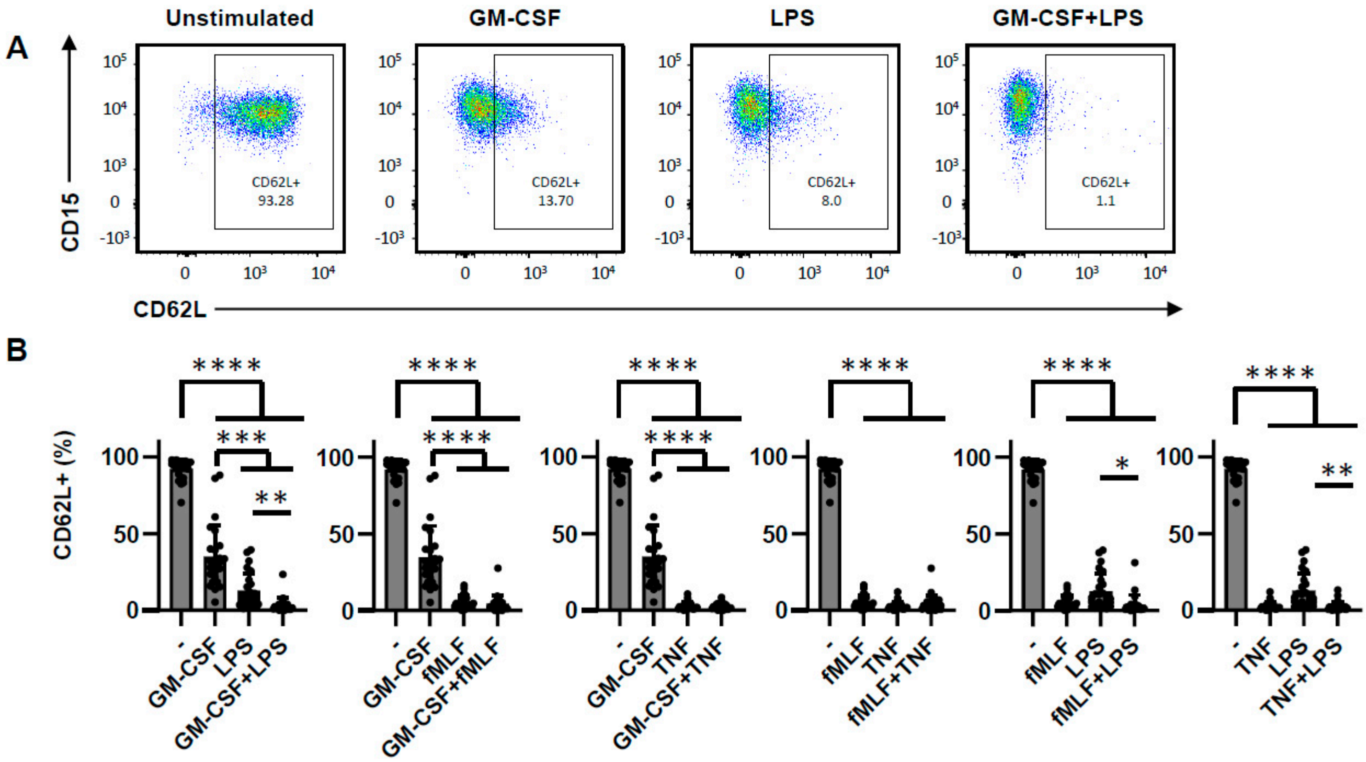

Figure S3. (**A**) Flow cytometry plot demonstrating gating strategy to determine CD62L membrane expression. (**B**) Neutrophils were cultured for 2 h in the absence or presence of different stimuli (GM-CSF (50 U/mL), LPS (10 ng/mL), fMLF (1 µM), and/or TNF (1 ng/mL)). CD62L membrane expression, expressed by percentages of CD62L+ neutrophils n = 20. Data are presented as mean ± SD. * $p < 0.05$, ** $p < 0.01$, *** $p < 0.001$, and **** $p < 0.0001$, One-way ANOVA.

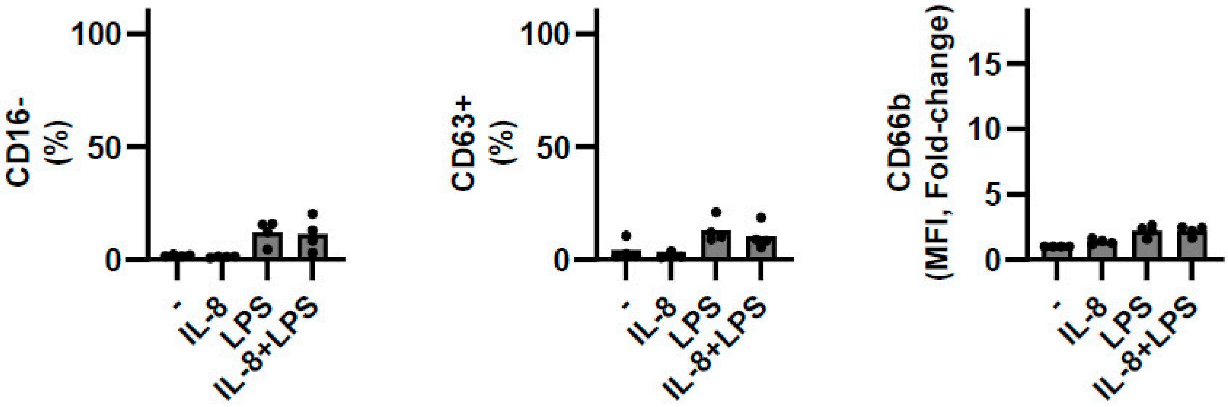

Figure S4. Neutrophils were cultured for 2 h in the absence or presence of LPS (10 ng/mL) and/or IL-8 (100 ng/mL)). CD16- and CD63+ membrane expression, expressed by percentages of CD16- and CD63+ neutrophils. CD66b membrane expression, as measured by changes in mean fluorescent intensity (MFI) of CD66b, expressed in fold-change compared to unstimulated. *n* = 4. Data are presented as mean ± SD.

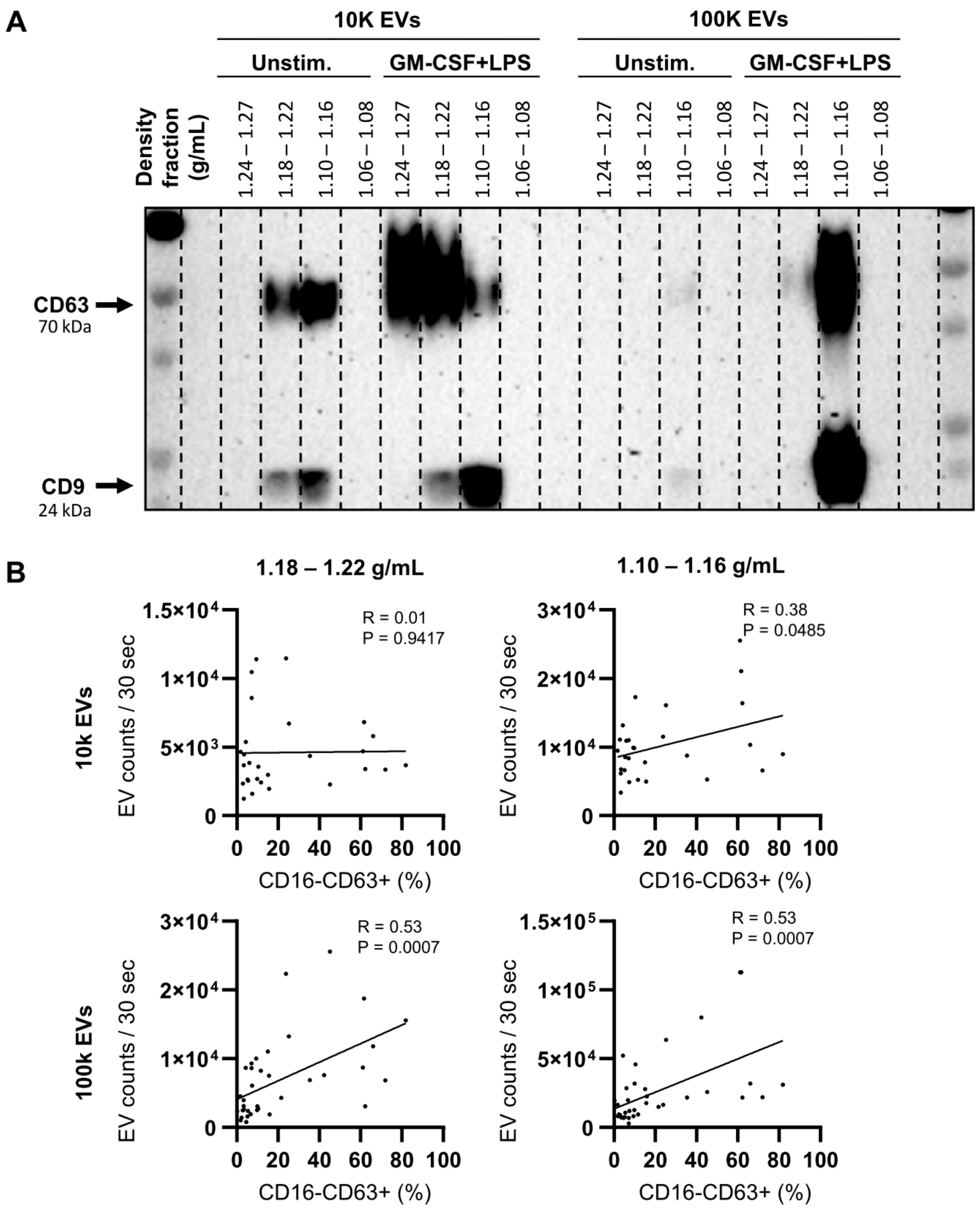

Figure S5. (**A**) Prolonged exposure of the western blot of Figure 6A of EVs pelleted at 10 kg and 100 kg and floated in a sucrose density gradient from neutrophils from a representative donor. Analy-sis is shown for CD9 and CD63 (tetraspan-ins; general EV-markers). (**B**) Linear relationships were determined between EV release shown in (Figure 6B) and full degranulation. Linear regression was applied to determine R.
